# Veterinary student wellbeing and its relationship with dog handling and fear responses during a clinical examination laboratory

**DOI:** 10.3389/fvets.2026.1910028

**Published:** 2026-08-20

**Authors:** Lindsay Nakonechny, Viera Pulver, Elpida Artemiou, Anastasia Chiara Stellato

**Affiliations:** 1 Department of Animal and Food Sciences, Texas Tech University, Lubbock, TX, United States; 2 Texas Tech School of Veterinary Medicine, Amarillo, TX, United States

**Keywords:** dog handling, dog welfare, fear, veterinary students, wellbeing

## Abstract

**Introduction:**

Veterinary care can be a significant source of fear and stress for dogs, and handling techniques play a key role in shaping these responses. Emerging evidence suggests that clinician mental wellbeing and stress can influence patient interactions. These challenges extend to veterinary students, who experience high stress during training and represent a critical population for understanding how these influences may emerge in clinical development. During a clinical examination laboratory, we aimed to assess (1) associations among veterinary students’ mental well-being and affective state, dog handling techniques, and dog fear responses, (2) whether dog fear responses were associated with students’ affective state, and (3) how students’ assessments of dog fear compared with investigator-rated fear scores.

**Methods:**

Third-year veterinary students (*n* = 66) performed physical examinations on dogs (*n* = 13) and communicated findings to a simulated client. Student mental well-being and affective state were measured using the WHO-5 and PANAS-SF, respectively. Following the laboratory, students rated the dog’s level of fear during the examination using a validated 5-item scale (0 = no fear, 4 = severe fear). Dog behavior and handling techniques were assessed from videos using one–zero sampling, with fear-related behaviors measured using an ethogram and the 5-item fear scale. Mixed regression models examined associations between student and examination factors, and dog fear outcomes.

**Results:**

Passive handling was associated with lower dog fear scores and fear-related behaviors; whereas more restrictive techniques, including standing restraint, abdominal lift, and muzzle hold, were associated with higher fear scores and behaviors (e.g., avoidance, reduced posture, lip/snout licking). Students assigned lower dog fear scores compared to the investigator (*p* < 0.001). Student mental well-being and affective state was not associated with handling techniques or dog fear outcomes; however, negative affect decreased post-laboratory (*p* < 0.001) and was associated with higher student-rated dog fear scores (*p* = 0.017).

**Discussion:**

These findings highlight the influence of handling techniques on dog fear responses during clinical training and suggest the need for enhanced education in behavior recognition. Although student well-being was not associated with dog handling, supporting resilience during training may foster positive clinical interactions throughout veterinary careers.

## Introduction

Routine veterinary visits can be a significant source of fear and stress for dogs, with signs of fear displayed as they enter the clinic (1), reside in the waiting room (2, 3), and during physical examination (1, 4). Fear can elevate the risk of aggression (5, 6), increasing the likelihood of injuries to patients, staff, and owners during routine handling. Consistent with this, surveys from North America and abroad report frequent animal-related injuries among veterinarians in various practice types (7–9).

To reduce fear responses in dogs and safety risks, it is recommended to use stress-reducing techniques during clinical care (10–12). These include minimizing physical restraint of dogs, using treats and distractions, performing examinations where the dog is most comfortable, allowing dogs to acclimate to the environment, and adjusting handling in response to dog behavior (10–13). Though limited, studies support these recommendations and show associations between stress-reducing techniques and reduced fear-related behaviors. Dogs show increased fear-related behaviors during physical examination phases that involve more physical contact and handling (4). Further, dogs handled using high levels of restraint (e.g., full-body restraint and muzzle-hold restraint) score higher on validated fear scales and display more fear-related behaviors, including escape attempts, vocalizations, avoidance, and postural reductions, compared to those handled using passive restraint (14). Whereas, when applying stress-reducing practices (e.g., use of anti-slip mats, minimal to no physical restraint, performing the examination where the dog is most comfortable), dogs showed lower stress scores and attenuated serum cortisol levels across four veterinary visits, compared to those handled using standard physical restraint, where the dog is held while standing or placed in lateral recumbency (15, 16). Having veterinary staff certified in a stress-reducing program (e.g., Fear Free®, Low Stress Handling®) has also been associated with reduced staff injuries from animal patients, compared to companion animal clinics where only some or no staff have such training (7). Though further research is needed to confirm these findings, studies indicate that using minimal restraint and other stress-reducing practices can promote human safety and reduce fear responses in dogs and stress during routine care.

As we gain more insights about the influence of different handling techniques on dog stress, research has begun to identify factors that influence the handling choices by veterinary staff. Low Stress Handling® guidelines emphasize the importance of adapting handling techniques according to the dog’s behavior during examinations, including adjusting and/or reducing the level of physical restriction if the dog resists the restraint for more than 3 s (10, 11, 17). Previous research identifies dog behavior as a primary driver of veterinary handling decisions; however, findings from previous survey studies suggest that, in practice, staff are more likely to use restrictive handling techniques (e.g., full-body restraint) when dogs are perceived as fearful or aggressive, compared to when they are calm (18, 19), contradicting recommendations. Qualitative research further highlights the range of handling strategies used in response to dog fear and aggression, including pausing and rescheduling appointments with pre-visit medications (e.g., trazadone), using chemical restraint, muzzles, towel wraps, or increased physical restraint (20).

Beyond patient behavior, multiple individual- and clinic-level factors also influence handling decisions. For instance, having a stress-reducing certification has been associated with veterinary staff using stress-reducing practices (21), whereas perceived time pressures and prioritization of examination completion have been identified as barriers (18). Veterinarian mental well-being has emerged as a potentially important, yet underexplored, influence on clinical behavior. Veterinary staff report high levels of stress, burnout, and psychological distress, with global estimates suggesting that approximately 37–73% experience high stress during their careers (22–27). Qualitative research suggests that these experiences may shape clinical care; veterinarians have described being less patient, shortening examinations, or reducing the use of stress-reducing techniques when experiencing high stress or compassion fatigue (20, 28). At the same time, some evidence suggests that poorer mental health may be associated with higher client satisfaction, potentially reflecting compensatory interpersonal strategies (26). These findings suggest that clinician well-being and stress may influence how veterinary staff interact with both clients and animal patients.

Importantly, these challenges are not limited to practicing veterinarians. Veterinary students also experience high levels of stress, anxiety, and depression due to demanding academic and clinical training environments (27–31). As stress often emerges during veterinary training and may persist into clinical practice, veterinary students represent a critical population for understanding how mental well-being may influence clinical behavior. While developing their clinical skills, students’ animal handling decisions may be more influenced by their mental state than those of experienced veterinarians, who have established examination routines. Exploring relationships among student well-being, animal handling techniques, and dog fear-related outcomes may therefore identify opportunities to support the development of technical competence and emotional regulation before students transition into clinical practice. Although previous research has examined the prevalence and determinants of veterinary student well-being, no studies have investigated the relationship between student well-being and clinical behavior, such as animal handling. Understanding how mental well-being and positive (e.g., excited, alert) and negative (e.g., distressed, nervous) emotions influence handling practices may provide insights into the development of clinical behaviors, and inform educational strategies that support both student development and optimal patient care.

Therefore, this study aimed to assess the following: whether veterinary students’ self-reported mental well-being and affective state are associated with handling techniques used, and with behavioral and physiological indicators of dog fear, if dogs’ fear responses and handling techniques used during examinations, in turn, influence students’ affective state, and how students score dog fear relative to experimenter-rated fear scores. To address these aims, we conducted an observational study during a veterinary student clinical communications laboratory and measured handling techniques and dog welfare indicators (fear-related behaviors, heart and respiration rate, body temperature) alongside students’ mental well-being and affective state. We hypothesized that lower student mental well-being and negative affective scores would be associated with more restrictive handling techniques during examinations, which would also relate to increased dog fear indicators.

## Materials and methods

The activities reported was approved by the Texas Tech University Institutional Animal Care and Use Committee (AUP#2024–1,500) and the Tech University Research Ethics Board (#IRB2025-504).

### Reflexivity statement

The research team held expertise in applied animal behavior, veterinary clinical education, and human-animal interaction research, which informed the study design and interpretation of the findings. The first author (LN) has over 12 years of experience assessing canine behavior in applied settings, including veterinary clinical care, and has experience teaching stress-reducing practices to veterinary students in companion animal handling laboratories at the Texas Tech University School of Veterinary Medicine (TTU SVM). LN was responsible for behavioral coding, and this background provided familiarity with dog fear-related behaviors and veterinary handling practices. To minimize potential bias, standardized behavioral definitions and coding procedures were established before data collection, statistical analyses followed a prespecified approach, and reflexivity was maintained through discussions among the research team to consider assumptions, sources of bias, and the interpretation of study findings.

### Participants

This study reports on a required veterinary integrated physical exam and communication skills laboratory for third-year veterinary students at the TTU SVM, located in Amarillo, Texas, United States. The laboratory, titled “Communicating the Physical Exam,” was part of the curriculum and was designed as a non-evaluative simulated client encounter involving a dog patient and a simulated client, defined as a trained volunteer portraying a dog owner. During the laboratory, students performed a routine physical examination on clinic dogs housed at TTU SVM while practicing physical examination skills and client communication.

The laboratory required students to apply the Calgary-Cambridge Guide framework (32) and the Value Matrix Tool (33) to collaboratively discuss and negotiate diagnostic options and associated costs, and to incorporate principles of spectrum of care (34) throughout the client interaction. Simulated clients completed a 1.2-h training session focused on the case scenario and communication skills grounded in the Calgary-Cambridge Guide and were instructed to follow a standardized script to support consistency across student encounters. The scenario involved a first-time client presenting a recently acquired stray dog for a wellness examination, with prompts related to vaccination, parasite prevention, and zoonotic disease concerns, including leptospirosis. The encounter also provided opportunities for students to demonstrate communication skills that support history-taking, client education, and shared decision-making.

During their first year in their veterinary curriculum, these students completed Fear Free Certified Professional® training and received instruction on applying stress-reducing practices. During their second year, they received training on how to conduct a routine physical examination on companion dogs. As part of the veterinary program, dogs are sourced from the Amarillo Animal Management and Welfare Shelter in Amarillo, Texas, prior to the start of the semester, housed at the TTU SVM for use in teaching activities, after which they are adopted out at the end of the semester. These dogs (*n* = 13; Table 1) participated in the laboratory and were up to date on vaccinations, had no current or chronic medical issues, and completed a behavior assessment by trained staff to verify absence of extreme fear or aggression upon exposure to unfamiliar people or exam-style handling. To mitigate stress levels, dogs were also administered an oral anxiolytic by facility staff prior to the laboratory, as per their animal care protocols. This protocol is part of the standard operating procedures for the facility and applies to every laboratory session in which teaching animals participate. The protocol includes oral administration of trazadone, 5–10 mg/kg, the night before the laboratory and the oral administration of trazadone, 5–10 mg/kg, and/or gabapentin, 10–20 mg/kg, 1–2 h prior to the start of the laboratory.

**Table 1:** Characteristics of dog subjects (*n* = 13) examined by veterinary students during a clinical examination laboratory (MN = male, neutered; FS = female, spayed).

Dog name	Sex	Age (months)	Breed	Weight (kgs)
Hera	FS	24	Heeler mix	25.2
Aurora	FS	22	Pitbull mix	24.5
Lila	FS	12	Pitbull mix	22.7
Mr. Man	MN	18	Husky mix	24.2
Squishy	MN	11	Pitbull mix	19.3
Chickpea	FS	15	American bulldog mix	24.1
Tuff	MN	17	Border collie mix	30.3
Bruno	MN	16	Anatolian shepherd	26.8
Goat	MN	24	Husky	23.6
Ruthless	FS	19	Pitbull	22.7
Copper	MN	27	Coonhound mix	26.6
Luigi	MN	12	Australian cattle dog mix	23.2
Brynlee	FS	36	Pitbull mix	24.9

The laboratory took place on August 15, 2025. This date falls at the beginning of the academic year for the third-year student cohort, prior to the start of their classes, as such, the students and dogs had no previous interactions. Students were scheduled to start at different times (8:30 a.m., 10:15 a.m., 1:30 p.m., and 3:15 p.m.), with each scheduled section taking approximately 1.5 h. Students were randomly assigned to each examination room with two to three students per room. They were also randomly assigned to a specific role, where they were either selected to be the examiner, who led and conducted the physical examination, or the handler, who performed all dog handling to facilitate the examination. If a third student was present, they refrained from all handling and instead engaged as the primary communicator, documenting and verbally communicating all examination findings for the instructors present. Two instructors were also present in each room to provide guidance and feedback on the students’ handling and client communication. The simulated client and dog patient remained in the examination room for the entire laboratory (1.5 h). Students were not formally assessed (e.g., did not receive a grade) for this laboratory.

Prior to the start of the laboratory, students completed a questionnaire that assessed their mental well-being and affective state. This questionnaire comprised of six questions, and was categorized into three sections: (1) dog handling: level of comfort handling calm, fearful, and aggressive dogs (using a 5-point Likert scale ranging from very uncomfortable to very comfortable), and number of total years handling dogs during routine examinations (including veterinary school); (2) students’ mental well-being over the past 2 weeks: using the 5-item World Health Organization-Five Well-Being Index (WHO-5); and (3) students’ positive (PA) and negative (NA) affective state: using the 20-item Positive and Negative Affect Schedule Short Form (PANAS-SF).

Upon entering their assigned consultation room, students were briefed by a clinician on the purpose and structure of the laboratory but were not provided with specific instructions on how to approach or handle the dog. The clinician then informed students of their role (i.e., handler or examiner), prompted the start of the physical examination, and allowed a maximum of 30 min for its completion. The structure of the examination followed their curricula training using a head-to-tail approach, though the order was not standardized. This involved starting with examining the dog’s head and moving towards the hind end, including assessment of the dogs’ vitals (i.e., temperature, heart and respiration rate). The dogs’ body temperature was measured using an otic thermometer (Braun Thermoscan, Kaz USA Inc.), heart rate was assessed via stethoscope (students used their personal stethoscope), and respiration rate was assessed via stethoscope or observing movement of the dog’s abdominal cavity. Once the examination was completed, the instructor proceeded to provide constructive feedback, and students completed a questionnaire comprised of two questions that was categorized into two sections: (1) students’ current affective state: using the PANAS-SF; (2) how fearful they perceived the dog to be during the examination: using a previously validated 5-point fear score (ranging from 0 to 4; 0 = no fear; 4 = severe fear; 4). Fear scores were derived from observations of dog body posture, avoidance and escape behaviors, and the occurrence of subtle stress indicators (e.g., yawning, lip licking, body shaking). Higher scores reflected greater reductions in dog posture, increased frequency of avoidance or escape responses, and subtle behaviors, and each score was paired with a demonstrative photo to guide assessments. For the full list of questions provided in the student pre- and post-laboratory questionnaires, see Supplementary File 1.

Within the examination room, an anti-slip mat was placed on the floor, where all examinations were performed. Students were provided with an otic thermometer and latex gloves and had access to restraint equipment during the examination, including muzzles and towels, if needed. If dogs showed signs of severe fear or aggression, based on the discretion of the TTU SVM animal caretakers, dogs were to be removed from the examination room for human safety and animal welfare purposes. All consultation rooms had the same dimensions (12 ft. by 12 ft) and were equipped with one-way observation windows, enabling faculty and staff to observe and oversee the examinations in real time without entering the rooms. The examinations were video recorded using HealthElevate Learning Space software (Elevate Healthcare, Florida, USA) from an integrated surveillance system installed in the consultation room ceilings. No dogs showed signs of severe fear or aggression during the physical examinations; thus, none were removed during this phase of the laboratory. Dogs were used across one to four laboratories (mean = 2.5 laboratories per dog).

### Behavioral data collection

Videos were uploaded to Noldus Observer XT 15 (Noldus, Wageningen, the Netherlands) to assess dog behavior and handling techniques applied by the assigned student handler (Table 2). An ethogram was developed from previous research exploring fear-related behavior during physical examination (Table 2; 4, 14, 35). Dog behaviors were recorded using one–zero sampling with 30-s sampling intervals. This sampling technique was selected to reduce inter-observer fatigue and maintain coding accuracy (36), as the examinations had relatively long durations (i.e., ranging from 4.0–20.1 min; median of 11 min). Behavior scoring was performed by the investigator and a trained observer. Inter-rater reliability was established through the investigator re-coding 10% of randomly selected videos, with a Kappa of 0.85 indicating excellent agreement. The investigator (LN) assigned the dog a fear score within each 30-s sampling interval using a 5-point scale (0–4; 0 = no fear; 4 = severe fear).

**Table 2:** Ethogram for behaviors measured during the physical examination.

Subject	Behavior	Description
Handler	Passive hold	Student has at least one hand on the dog’s body.
	Standing restraint	Dog is standing and student’s forearm or arm is holding the dog’s head and/or neck, and the student’s other arm is placed on another part of the dog’s body.
	Full-body restraint	Dog is placed laterally on its side, and the student holds at least one of the dog’s legs, allowing little to no movement.
	Abdominal lift	Dog is lifted vertically by the student onto its back legs with its abdomen exposed.
	Rear restraint	Student holds the dog’s hind limbs and/or back end with their arms.
	Muzzle hold	Student grasps the dog’s muzzle by placing one of their hands around the muzzle.
	Chin hold	Student places at least one hand underneath the dog’s chin.
	Collar hold	Student holds the dog’s collar with at least one hand.
Dog	Reduced posture	Head low, ears sideways, down or pinned back, tail lowered or tucked between bent hind legs, either still or wagging.
	Body shaking	Lateral, side to side rotation of the body about the central axis, with shaking of the fur.
	Lip/snout licking	Portion of the tongue moves along the upper lip.
	Yawning	Wide opening of the mouth.
	Vocalizing	Barking, growling, whining, yelping.
	Elimination	Dog eliminates their bladder or bowels.
	Avoidance	Dog attempts to avoid the student’s touch by moving in any direction away from the student. This includes movement of the dog’s head, body, and/or limbs.
	Escape	Dog successfully removes their body out of the student’s physical restraint.

### Data management

Analyses were performed using Stata Statistical Software v.17.0 (StataCorp., College Stations, TX, USA). A total of 14 variables were included in the analyses, including handler mental well-being (WHO-5 score), handler pre-laboratory positive affect (PA) and negative affect (NA), handler-perceived dog fear score, handling techniques, the proportion of passive (vs. non-passive) handling techniques used (i.e., passive restraint ratio), and examination duration.

For student well-being, the raw scores from the WHO-5 measure (0 to 25) were converted to percentages by multiplying the raw score by 4 (scores ranged from 0 to 100; 0 = worst possible, 100 = best possible mental well-being), as per Bech et al. (37). Students’ pre-laboratory NA and PA were evaluated independently, as per Watson et al. (38); PA and NA scores ranged from 10 to 50, with higher scores indicating greater positive or negative affect, respectively.

Average dog fear score was calculated by averaging dog fear scores assigned by the investigator across all 30-s sampling intervals within each examination (Table 3). This approach allowed dog fear to be assessed at the examination-level while accounting for variation in examination duration. This variable was retained as a continuous measure.

**Table 3:** Mixed linear regression results for factors associated with average dog fear score with dog ID included as a random effect (*n* = 33 examinations; *R*^2^ = 0.254).

Model outcome	Variable	Coefficient	95% CI	*P-*value
Average dog fear score				
Passive restraint ratio	−	−1.81	−2.89, −0.74	0.001

For interval-level analyses, dog fear scores were dichotomized into none/mild fear (scores 0–1) and moderate/extreme fear (scores 2–4). This was necessary because the outcome variable did not meet the assumptions required for a linear model at the interval level (e.g., non-independence of observations within exam, heteroscedasticity), and because of sparse observations in the highest fear category (score 4). Handling techniques (i.e., passive hold, full-body restraint, abdominal lift, standing restraint, rear restraint, muzzle hold, chin hold, and collar hold) were recorded during each 30-s sampling interval as binary variables (0 = not observed, 1 = observed). A passive restraint ratio was calculated to quantify the proportion of passive holds used during an examination, relative to other handling techniques (i.e., full-body restraint, abdominal lift, standing restraint, rear restraint, muzzle hold, chin hold, and collar hold). This variable was calculated by dividing the number of 30-s sampling intervals in which a passive hold was observed by the total number of scorable intervals within a given examination. For example, if a passive hold was observed during 6 out of 15 sampling intervals, the passive restraint ratio was 0.40. Higher values indicate greater use of passive holds by the handler. This variable was retained as a continuous measure, representing the relative use of passive holds versus other handling techniques.

### Comparing student measures across laboratory participation

Paired t-tests were used to compare students’ pre- and post-laboratory NA and PA scores, with normality assessed visually using histograms. Wilcoxon signed-rank tests were used to compare student-perceived dog fear scores with investigator-rated fear scores, given the ordinal nature of the dog fear scale.

### Risk factors analysis

A series of mixed regression models were developed to examine factors associated with the following outcomes: dog fear scores, dog fear-related behaviors (i.e., reduced posture, body shaking, lip/snout licking, yawning, vocalizing, elimination, avoidance, and escape), passive restraint ratio, and handler post-laboratory NA and PA.

For logistic models, results were reported as the odds ratios (OR), where OR > 1 indicates higher odds (possible causal effect) and OR < 1 indicates lower odds (possible protective effect). Depending on the level of analysis, random effects were specified to account for non-independence in the data. For interval-level data (30-s observations), exam was included as a random effect to account for the non-independence of intervals sampled within the same examination. For examination-level data, dog was included as a random effect to account for the repeated use of the same dogs across multiple laboratory sessions. Only data from students assigned to the handler role, including their mental well-being (WHO-5 score) and pre- and post-laboratory affective state (NA and PA scores), were included in the regression analyses. As only the handler was directly responsible for applying handling techniques during the examination, only handler-level variables were included in the regression analyses to ensure that predictors (e.g., handling techniques) were appropriately aligned with outcome variables (e.g., dog fear responses).

### Factors associated with average dog fear score

A mixed linear regression model was used to evaluate factors associated with average dog fear score at the examination-level (Table 3). The following explanatory variables were assessed: handler mental well-being, handler pre-laboratory NA, handler pre-laboratory PA, examination duration, passive restraint ratio, and handler-perceived dog fear score.

### Factors associated with moderate/extreme dog fear

A mixed logistic regression model was used to evaluate factors associated with moderate/extreme dog fear scores (i.e., scores of 2–4) at the interval-level (Table 4). Handling techniques were assessed as explanatory variables (i.e., passive hold, full-body restraint, abdominal lift, standing restraint, rear restraint, muzzle hold, chin hold, collar hold), which were binary variables (0 = not observed, 1 = observed).

**Table 4:** Mixed logistic regression model for handling techniques associated with dog fear scores, with examination ID included as a random effect (*n* = 627 sampling intervals).

Model outcome	Variable	OR	95% CI	*P-*value
Average dog fear score^a^				
Passive hold	Yes vs. No	0.18	0.09, 0.38	<0.001
Standing restraint	Yes vs. No	2.52	1.22, 5.20	0.012
Muzzle hold	Yes vs. No	80.90	8.39, 780.37	<0.001

^a^ The intraclass correlation coefficient (ICC) = 0.51, 95% CI: 0.34–0.67.

### Factors associated with individual dog fear-related behaviors

Separate mixed logistic regression models were conducted for each dog fear-related behavior (i.e., reduced posture, body shaking, lip/snout licking, yawning, vocalization, elimination, and avoidance) at the interval-level (Table 5). Each behavior was coded as a binary outcome (0 = not observed, 1 = observed) for each 30-s sampling interval, within an examination. For each model, handling techniques were included as explanatory variables (i.e., passive hold, full-body restraint, abdominal lift, standing restraint, rear restraint, muzzle hold, chin hold, collar hold).

**Table 5:** Mixed logistic regression models for handling techniques associated with dog fear behaviors, with examination ID included as a random effect (*n* = 627 sampling intervals).

Model outcome	Variable	OR	95% CI	*P-*value
Reduced posture^a^				
Abdominal lift	Yes vs. No	38.87	3.64, 414.64	0.002
Standing restraint	Yes vs. No	8.35	4.05, 17.23	<0.001
Lip/snout licking^b^				
Abdominal lift	Yes vs. No	15.18	2.44, 94.54	0.004
Avoidance^c^				
Passive hold	Yes vs. No	0.23	−0.11, 0.46	<0.001
Standing restraint	Yes vs. No	2.91	1.46, 5.81	0.002
Muzzle hold	Yes vs. No	21.61	5.13, 92.95	<0.001
Escape^d^				
Muzzle hold	Yes vs. No	13.68	1.30, 144.09	0.029

^a^ The intraclass correlation coefficient (ICC) = 0.68, 95% CI: 0.51–0.81. ^b^ ICC = 0.55, 95% CI: 0.30–0.78. ^c^ ICC = 0.49, 95% CI: 0.32–0.66. ^d^ ICC = 0.56, 95% CI: 0.12–0.93.

### Factors associated with passive restraint ratio

A mixed linear regression model was used to evaluate factors associated with the proportion of passive holds used during examinations, relative to other handling techniques used. Explanatory variables assessed included: handler mental well-being, handler pre-laboratory NA, handler pre-laboratory PA, examination duration, and handler-perceived dog fear score.

### Factors associated with handler post-laboratory affective state

Separate mixed linear regression models were conducted to evaluate factors associated with handler post-laboratory NA and PA (Table 6). Explanatory variables assessed included: handler mental well-being, examination duration, handler-perceived dog fear score, and passive restraint ratio.

**Table 6:** Mixed linear regression model for factors associated with handler post-laboratory NA, with dog ID included as a random effect (*n* = 29 examinations; *R*^2^ = 0.091).

Model outcome	Variable	Coefficient	95% CI	*P-*value
Handler post-laboratory NA				
Handler dog fear score	Score 0	Referent	–	0.017
	Score 1	−2.51	−4.72, −0.31	0.026
	Score 2	−2.60	−6.61, 1.41	0.20

### Model building

Linear relationships between continuous explanatory variables (i.e., WHO-5, pre-lab NA and PA, examination duration) and outcome variables were assessed using locally weighted regression curves and testing significance of the quadratic term (39). As these variables did not demonstrate linear relationships, and transformations did not improve linearity, they were subsequently categorized for inclusion in the regression models (39). The WHO-5 was categorized using a recommended threshold (≤50 vs. >50), where students scoring ≤50 were considered at risk for reduced well-being (34). Affective state (PA and NA) and examination duration were categorized using sample distributions (mean and median values) to create balanced group sizes and support model stability (39). Affective state variables were categorized as ‘low’ and ‘high’ using the mean of pre- and post-laboratory scores (PA: low = ≤34 vs. high = > 34; NA: low = ≤15 vs. high = > 15), and examination duration (minutes) was dichotomized at the median value (≤11 vs. >11 min).

Correlations between all explanatory variables were tested, and none were found to have a correlation coefficient of >70%. Univariate analysis was performed to test the association of each independent variable against each outcome variable and variables with a liberal *p*-value of *p* ≤ 0.20 were retained for model building (39). Each multivariable model was then built using a forward stepwise selection process where only significant explanatory variables (*p* < 0.05) were retained in the full model. Confounders were based on biological plausibility and were identified as an explanatory variable that caused >30% change in the coefficient of another variable in the model. This threshold was chosen as a conservative criterion for identifying covariates with substantial influence on other variables (39). When confounders were identified they were included in the model. Biologically plausible two-way interaction terms were tested between variables in the final the main effects model; no significant interactions were identified. The risk factors generated from these models are reported as statistical associations without implying causality or temporal direction.

## Results

Data were analyzed from 66 of the 97 third-year veterinary students who completed the required laboratory, including 33 handlers and 33 examiners. Data from the 31 students assigned to the communicator role, who were not involved in dog handling or examination, were excluded from analyses.

The mean (±SD) frequency of handling technique used across all 33 examinations was highest for standing restraint (15.1 ± 7.6), followed by passive hold (4.6 ± 3.6), muzzle hold (0.9 ± 1.4), collar hold (0.6 ± 1.9), chin hold (0.5 ± 0.8), abdominal lift (0.3 ± 0.7), rear restraint (0.3 ± 0.8), and full-body restraint (0.3 ± 0.9). Students’ level of comfort with dog handling differed according to dog demeanor. Most students reported being very or slightly comfortable handling calm dogs (62/66; 93.9%), compared with fearful (57/66; 86.4%) or aggressive dogs (32/66; 48.5%; see Supplementary File 2: Table 1). Most students agreed or strongly agreed that they were more comfortable handling medium/large dogs compared with small dogs (48/65; 73.9%; see Supplementary File 2: Table 2). In contrast, fewer students agreed or strongly agreed that they were more comfortable handling cats than dogs (17/65; 26.2%), while approximately half agreed or strongly agreed that they were more comfortable handling livestock than dogs (33/65; 50.8%; see Supplementary File 2). On average, students reported 6.5 ± 3.2 years of experience handling dogs during routine examinations, including their experience obtained during veterinary school. Descriptive statistics for student mental wellbeing and affective state before and after the laboratory are presented in Supplementary File 2: Table 3.

### Dog information and physiological measures

A total of 13 dogs were included in this study, with ages ranging from 11 to 36 months (19.5 ± 7.1 months). All dogs weighed >19kgs and the majority were mixed breeds (Table 2). Regarding the physiological measures, students were expected to assess and verbally report body temperature, heart rate, and respiration rate as part of the physical examination; however, only a subset of students verbally reported these measures during the video-recorded sessions. Thus, these variables were not included in further analyses due to an insufficient number of observations. The average dog body temperature (*n* = 3) was 100.8 °F ± 1.1 (range: 99.0–103.0 °F), the average heart rate (*n* = 12) was 91.6 ± 19.3 beats/min (range: 64–120 beats/min), and the average respiration rate (*n* = 3) was 23.3 ± 12.1 breaths/min (range: 12–36 breaths/min).

When combining handler and examiner students, there was no significant difference in PA between pre- and post-laboratory scores [mean difference = −0.31, 95% CI: −1.61 to 0.99; *t*(57) = − 0.48, *p* = 0.633]. In contrast, students’ NA was significantly lower post-laboratory compared to pre-laboratory [mean difference = 2.56, 95% CI: 1.25 to 3.87; *t*(58) = 3.90, *p* < 0.001]. This corresponds to a moderate effect size (Cohen’s d = 0.51), indicating a meaningful reduction in NA following the laboratory session. When examined by individual role, similar patterns were observed, with no significant difference in PA between pre- and post-laboratory scores (handler: *p* = 0.908; examiner: *p* = 0.592); however, NA was significantly lower post-laboratory compared to pre-lab (handler: *p* = 0.003; examiner: *p* = 0.025).

### Comparing experimenter and student ratings of dog fear

Based on the Wilcoxon signed-rank test, student-perceived dog fear scores differed significantly from experimenter-rated fear scores when data from handler and examiner students were combined together (z = 3.66, *p* < 0.001). When examined by individual student role, similar patterns were observed for the handler (*p* < 0.001) and examiner (*p* = 0.001) students. In the majority of paired observations (25/33), experimenter ratings of fear exceeded student ratings, indicating that students generally underestimated the dogs’ level of fear during examination.

### Risk factors

#### Average dog fear score

Linear regression results revealed that a higher passive restraint ratio was associated with lower average dog fear scores (Table 3). The following variables were not associated with average dog fear score: handler mental well-being, handler pre-laboratory NA, handler pre-laboratory PA, and examination duration.

#### Dog fear score

Logistic regression results revealed that passive hold was associated with lower odds of dogs being scored as showing moderate/extreme fear (i.e., fear scores of 2–4); whereas standing restraint or muzzle hold were associated with high odds of dogs showing moderate/extreme fear (Table 4). Although muzzle holds were used infrequently, this handling technique was associated with higher odds of dogs showing moderate/extreme fear. The following variables were not associated with dog fear score: full-body restraint, abdominal lift, rear restraint, chin hold, and collar hold.

#### Dog fear-related behaviors

Logistic regression results revealed that abdominal lift or standing restraint were associated with higher odds of reduced posture and lip/snout licking (Table 5). Passive hold was associated with lower odds of avoidance, whereas standing restraint and muzzle hold were associated with higher odds of avoidance and escape (Table 5). Regression results did not reveal any significant associations between handling techniques and the following dog fear-related behavior outcomes: body shaking, yawning, vocalizing, and elimination.

#### Passive restraint ratio

None of the following variables were significantly associated with passive restraint ratio: handler mental well-being, handler pre-laboratory NA, handler pre-laboratory PA, handler-perceived dog fear, and examination duration.

#### Handler post-laboratory affective state

Handler students’ perceiving the dog to have a fear score of 2 or 1 (vs 0) was associated with lower post-laboratory NA (Table 6). The following variables were not associated with handler post-laboratory NA: passive restraint ratio, and examination duration. Handler mental wellbeing was identified as a confounder on handler post-laboratory lab NA and thus was retained in the model. No predictors were identified for handler post-laboratory PA.

## Discussion

### Handling techniques and dog fear

The current study found that the application of passive handling by veterinary students was associated with lower dog fear scores and fewer fear-related behaviors; whereas the application of more restrictive handling techniques, including standing restraint, abdominal lift, and muzzle-hold, were associated with increased dog fear scores and several fear-related behaviors (i.e., avoidance, escape, reduced posture, and lip licking). Findings align with previous experimental studies evaluating stress-reducing handling approaches, which have shown that lower levels of physical restraint are associated with fewer indicators of dog stress (4, 40, 41, 14–16). The application of restrictive techniques may increase perceived threat by limiting the dog’s movement and reducing opportunities for avoidance or control, thereby heightening animal fear responses (10, 42). Muzzle hold was also associated with increased escape behavior, and similarly, when evaluating dog behavioral and physiological responses to different handling techniques, Cisneros et al. (14) found increased escape attempts and fear scores when a muzzle hold was applied, compared to passive restraint. Handling that restricts the muzzle in this way may be particularly aversive as it limits the dog’s ability to perform normal oral behaviors [e.g., panting, mouth opening; (17, 43)]. Further, the relationship between handling and dog fear is likely dynamic, where handling decisions both influence and are influenced by the dog’s behavior. For example, restrictive handling techniques may elicit elevated fear responses, which may prompt the application of more restraint to maintain control. This bidirectional interaction highlights the complexity of human–animal interactions during clinical examinations and the importance of adapting handling approaches based on the dog’s behavioral responses.

Students also perceived dog fear to be lower during examination, compared to the experimenter, consistent with previous research that highlights the difficulty veterinary students and professionals have in recognizing subtle indicators of fear in dogs, such as yawning and paw-lifting (44). Tami and Gallagher (45) similarly found few differences in the ability to interpret dog behavioral states, including fear and aggression, between veterinarians and dog owners, suggesting that veterinary training may not confer greater accuracy in recognizing dog behavior. Poor recognition of subtle fear indicators may lead to the use of handling techniques that are not appropriately adapted to the dog’s behavior, potentially increasing dog fear and risk of injury, contrary to stress-reducing handling recommendations (2, 10, 17). Assessing behavior is not only important for minimizing animal stress and reducing injury during veterinary care, but also for supporting accurate diagnoses (e.g., pain-related behavior), consultation, and treatment for behavioral concerns (46, 47). Having difficulty recognizing animal fear may be attributed to limited training and education in animal behavior in veterinary colleges across the United States (46, 48). Several studies indicate that veterinarians report limited skills and confidence in behavioral medicine and seek continuing education to strengthen their knowledge of animal behavior (49, 50). This gap in knowledge may also negatively affect veterinary wellbeing when in practice, as a qualitative study found that perceived deficiencies in knowledge and confidence when handling fearful or aggressive dogs can contribute to early-career stress and burnout (20).

When transitioning from academic learning to hands-on practice, veterinary students often lack confidence and face challenges with technical competency, time management, and emotional regulation (51). Students may also have challenges managing competing demands during clinical examinations, including client communication, animal handling, and decision-making. Given that one of the laboratory objectives was effective client communication, it is possible that students prioritized interactions with the client, and paid less attention to the dog’s behavior, contributing to the differences observed between student and experimenter dog fear scores.

To better prepare students for clinical realities and enhance essential professional skills (e.g., client communication), early integration of skill development within veterinary curricula, reinforced through experiential learning, is recommended (52). Exposure to diverse, real-world scenarios allow students to practically apply and refine their skills and build resilience through constructive instructor feedback (53, 54). These recommendations may also be applicable for enhancing students’ animal handling skills. Integrating animal behavior education early and consistently throughout the curriculum, alongside in-depth instruction in stress-reducing handling techniques and opportunities to navigate realistic clinical scenarios (e.g., managing fearful or aggressive patients), may enhance student competence and confidence in applying appropriate handling strategies. Such training may ultimately promote safer, lower-stress clinical interactions, benefiting both animal welfare and veterinary personnel.

### Student wellbeing and affective state

A significant reduction in students’ negative affect was observed following the laboratory session, while positive affect remained unchanged. This suggests that the clinical laboratory experience may have alleviated anticipatory stress or anxiety without substantially affecting positive emotional states. The decrease in negative affect may also reflect students’ appraisal of their performance during the examination, as emotions in educational contexts are shaped by perceived competence and success in achievement-related tasks (55). Interestingly, post-laboratory handler negative affect was associated with perceived dog fear score; specifically, negative affect decreased when handlers perceived dogs to be mildly fearful during the examination, compared to not fearful at all. This may reflect an achievement-related emotional response (55), whereby successfully completing an examination on a fearful animal patient contributed to students’ perceived competence, consistent with clinical education research showing that simulated clinical encounters can support the self-efficacy of students in healthcare (56, 57).

Student well-being and pre-lab affective state was not associated with handling techniques applied or dog fear measured. This finding contrasts with qualitative and survey-based studies in practicing veterinarians, where mental well-being has been shown to influence animal handling (18, 20, 28). One possible explanation for these findings is that the current study was conducted in a controlled academic setting, which does not capture the complexity and pressures of clinical practice, where veterinarians are exposed to cumulative workplace and occupational stressors, such as high workloads, long hours, and challenging interactions with clients and colleagues (58–60) that may amplify the influence of mental well-being on handling decisions. As such, the absence of these associations in the current study may suggest that broader workplace and occupational stressors encountered in clinical practice play a role in shaping the relationship between mental wellbeing and clinical behavior observed in practicing veterinarians. It remains important to introduce concepts related to stress, burnout, compassion fatigue, compassion satisfaction, and coping strategies early in veterinary education so that students are better equipped to manage these stressors upon entering clinical practice. Further research is needed to substantiate these findings and to explore how veterinary students currently cope with stressors, as well as their understanding of burnout and stress, to inform targeted resiliency interventions.

Most students scored above the WHO-5 risk threshold (>50), with scores concentrated toward the upper end of the scale, suggesting that students entering their third year were not at elevated risk for reduced well-being at this point in the academic year; only 4 of 33 handlers (12.1%) and 7 of 33 examiners (21.2%) had scores at or below 50. This pattern may reflect the timing of the laboratory, which occurred at the start of the fall semester before the cumulative demands of the clinical year had fully accrued. The timing of assessments within the veterinary curriculum may influence when student well-being is most likely to influence clinical performance. Future research assessing well-being and affect longitudinally across the academic year could help identify when these factors are most likely to influence students’ clinical performance, informing the timing and targeting of curricular supports. Further, these self-reported measures may be subject to response bias, particularly in the current laboratory setting where students may have prioritized task completion instead of reflecting on each prompt. Also, despite the laboratory not being formally evaluated it may have prompted more socially desirable responses (61).

### Implications for veterinary student education

These findings have several practical implications for veterinary education. The associations between handling techniques and dog fear supports continued emphasis on stress-reducing handling practices, including passive handling and adapting handling techniques to dog behavior. The observed gap between student perceived and experimenter-rated dog fear highlights the need for additional student training in behavioral observation and interpretation, particularly in identifying subtle indicators of fear. Incorporating enhanced education and practical training opportunities on dog behavior, fear assessment, and stress-reducing practices into veterinary curricula may improve students’ ability to apply handling practices that optimize dog welfare and handler safety. Although student mental well-being was not associated with handling and dog fear outcomes in this study, the broader literature suggests that mental health affects clinical care in practice (20, 28). As such, early integration of mental health awareness, resilience training, and effective coping strategies within veterinary education may help prepare students to navigate these challenges. Supporting student well-being is therefore important for promoting patient-centered care, supporting individual well-being, and building a sustainable veterinary workforce as students transition into clinical practice.

### Limitations

Several limitations should be considered when interpreting these findings. This observational study was conducted in a simulated academic clinical environment, which does not reflect real-world veterinary practice, particularly with respect to cumulative workplace-related stress and clinical pressures. Thus, these results are not generalizable to veterinary populations. Prior to the laboratory, dogs underwent behavioral assessments to limit risks to veterinary students, and were administered anxiolytic medications (i.e., trazadone, gabapentin) which have been shown to decrease behavioral indicators of stress in dogs during veterinary visits (62), and in hospitalized dogs (63). Thus, the use of these medications may have reduced the frequency and/or range of behavioral fear responses observed, limiting generalizability to broader dog patient populations. Several dogs within the sample were also pit bull-type breeds or mixes, which may have influenced students’ handling approaches. Therefore, students may have approached some dogs with heightened perceived risk due to breed behavioral stereotypes independent of the dogs’ observed behavior potentially confounding the observed relationship between student perceptions and clinical outcomes. Future research should include a more diverse range of dog breeds and sizes to determine whether these findings are consistent across a broader patient population.

The large effect sizes and wide confidence intervals observed for certain handling techniques, particularly muzzle hold and abdominal lift, should be interpreted with caution. These techniques were infrequently used, but when they occurred, dogs exhibited elevated fear levels. This pattern suggests potential instability in these estimates due to sparse data. Further, students’ handling decisions may have also been influenced by the presence of supervising clinicians in the examination rooms.

Physiological measures were collected as part of the study protocol; however, these data were inconsistently recorded by students and were therefore not included in the regression analyses. Consequently, the findings are based primarily on behavioral indicators of dog fear. Future studies should incorporate standardized collection of physiological data alongside behavioral observations to provide a more comprehensive assessment of dog responses during veterinary examinations.

An *a priori* sample size calculation was not performed because the sample was determined by existing class enrollment rather than a predetermined estimate of the effect size needed to detect a meaningful difference. As a result, the study may have been underpowered to detect associations between student mental well-being, affect, handling techniques, and dog fear. Furthermore, several continuous explanatory variables were categorized when analyses indicated non-linear relationships with outcome variables. Although this approach allowed non-linear associations to be incorporated and improved model interpretability, categorization may have reduced sensitivity to detect more subtle relationships. Thus, the models should be interpreted as exploratory and hypothesis-generating. The observational nature of this study also limits conclusions regarding causality and temporal relationship between dog fear responses and handling techniques. Although fear-related behaviors and handling techniques were recorded, the sampling structure and limited number of fear-related behaviors per examination did not allow reliable evaluation of within-examination changes in fear responses. Future studies using designs with more frequent or continuous behavioral sampling should investigate temporal patterns to determine whether changes in dog fear precede changes in handling techniques, or whether handling practices influence subsequent dog fear responses.

## Conclusion

During a clinical communication laboratory for third-year veterinary students, handling techniques were associated with dogs’ fear responses: greater use of passive handling corresponded with lower fear scores and fewer fear-related behaviors, whereas more restrictive techniques corresponded with increased fear responses, consistent with current handling recommendations. The relationship between handling and dog fear is likely dynamic, where handling decisions both influence and are influenced by the dog’s behavior. Student mental wellbeing and affective state were not associated with handling practices or dog fear outcomes in this educational setting, which may reflect limited variability in psychological measures and the structured, supervised nature of the laboratory. However, broader evidence suggests that under real-world clinical conditions, workplace stressors may shape how handling decisions are made, highlighting the importance of introducing resilience-building, coping strategies, and mental health awareness early in veterinary training to better prepare students for these challenges. Students also tended to rate dog fear as lower compared to experimenter scores, indicating a potential gap in veterinary student curriculum. These findings demonstrate relationships between handling techniques and dog fear, and the importance of integrating stress-reducing handling and animal behavioral training within veterinary education. Such efforts may support both animal welfare and the development of competent, adaptable veterinarians.

## Data Availability

The raw data supporting the conclusions of this article will be made available by the authors, without undue reservation.
