# Supplementary material for: Veterinary student wellbeing and its relationship with dog handling and fear responses during a clinical examination laboratory
Source: Front Vet Sci. 2026 Aug 20;13:1910028. doi: 10.3389/fvets.2026.1910028 (PMC13538008; doi:10.3389/fvets.2026.1910028)
Supplement: Supplementary file 1 [file Supplementary_file_1.DOCX]

Supplementary Material

**Pre-lab Questionnaire**

**1. What is your full name?**

________________________________________________________________

**2. For the purposes of this question, calm, fearful, and aggressive are defined in the following ways:**

- **Calm:** Relaxed, no signs of aggression or fear-related behaviors
- **Fearful:** Showing fear-related behaviors, such as lowered posture, ears back, tail tucked, whimpering or whining, shaking or trembling, attempts to hide or escape
- **Aggressive:** Showing fear-related behaviors while showing aggression, such as baring teeth, attempting to bite, growling, lunging

**Please indicate your level of comfort handling the following dogs during a routine health examination (select one per row):**

|  | **Very Uncomfortable** | **Slightly Uncomfortable** | **Neither Comfortable nor Uncomfortable** | **Slightly Comfortable** | **Very Comfortable** | **Prefer not to say** |
| --- | --- | --- | --- | --- | --- | --- |
| **Calm dog** | ☐ | ☐ | ☐ | ☐ | ☐ | ☐ |
| **Fearful dog** | ☐ | ☐ | ☐ | ☐ | ☐ | ☐ |
| **Aggressive dog** | ☐ | ☐ | ☐ | ☐ | ☐ | ☐ |

**3. Please report your level of agreement with the following questions (select one per row):**

|  | **Strongly agree** | **Agree** | **Neutral** | **Disagree** | **Strongly disagree** | **Prefer not to say** |
| --- | --- | --- | --- | --- | --- | --- |
| **I am more comfortable handling medium/large dogs (e.g., 35 lbs or greater) compared to small breed dogs (e.g., under 35 lbs)** | ☐ | ☐ | ☐ | ☐ | ☐ | ☐ |
| **I am more comfortable handling cats compared to dogs** | ☐ | ☐ | ☐ | ☐ | ☐ | ☐ |
| **I am more comfortable handling livestock (e.g., cattle, horses) compared to dogs** | ☐ | ☐ | ☐ | ☐ | ☐ | ☐ |

**4. How many years of veterinary clinical experience handling dogs during routine examinations and procedures do you have (including your time in veterinary school)?**

________________________________________________________________

**5. Please respond to each item below by marking one box per row, regarding how you felt in the last two weeks (select one per row**):

|  | **All of the time** | **Most of the time** | **More than half the time** | **Less than half the time** | **Some of the time** | **At no time** |
| --- | --- | --- | --- | --- | --- | --- |
| **1. I have felt cheerful in good spirits.** | ☐ | ☐ | ☐ | ☐ | ☐ | ☐ |
| **2. I have felt calm and relaxed.** | ☐ | ☐ | ☐ | ☐ | ☐ | ☐ |
| **3. I have felt active and vigorous.** | ☐ | ☐ | ☐ | ☐ | ☐ | ☐ |
| **4. I woke up feeling fresh and rested.** | ☐ | ☐ | ☐ | ☐ | ☐ | ☐ |
| **5. My daily life has been filled with things that interest me.** | ☐ | ☐ | ☐ | ☐ | ☐ | ☐ |

**6. This scale consists of a number of words that describe different feelings and emotions. Read each item and indicate to what extent you feel this way right now, that is, at the present moment (select one per row):**

|  | **Very slightly or not at all** | **A little** | **Moderately** | **Quite a bit** | **Extremely** |
| --- | --- | --- | --- | --- | --- |
| **1. Interested** | ☐ | ☐ | ☐ | ☐ | ☐ |
| **2. Distressed** | ☐ | ☐ | ☐ | ☐ | ☐ |
| **3. Excited** | ☐ | ☐ | ☐ | ☐ | ☐ |
| **4. Upset** | ☐ | ☐ | ☐ | ☐ | ☐ |
| **5. Strong** | ☐ | ☐ | ☐ | ☐ | ☐ |
| **6. Guilty** | ☐ | ☐ | ☐ | ☐ | ☐ |
| **7. Scared** | ☐ | ☐ | ☐ | ☐ | ☐ |
| **8. Hostile** | ☐ | ☐ | ☐ | ☐ | ☐ |
| **9. Enthusiastic** | ☐ | ☐ | ☐ | ☐ | ☐ |
| **10. Proud** | ☐ | ☐ | ☐ | ☐ | ☐ |
| **11. Irritable** | ☐ | ☐ | ☐ | ☐ | ☐ |
| **12. Alert** | ☐ | ☐ | ☐ | ☐ | ☐ |
| **13. Ashamed** | ☐ | ☐ | ☐ | ☐ | ☐ |
| **14. Inspired** | ☐ | ☐ | ☐ | ☐ | ☐ |
| **15. Nervous** | ☐ | ☐ | ☐ | ☐ | ☐ |
| **16. Determined** | ☐ | ☐ | ☐ | ☐ | ☐ |
| **17. Attentive** | ☐ | ☐ | ☐ | ☐ | ☐ |
| **18. Jittery** | ☐ | ☐ | ☐ | ☐ | ☐ |
| **19. Active** | ☐ | ☐ | ☐ | ☐ | ☐ |
| **20. Afraid** | ☐ | ☐ | ☐ | ☐ | ☐ |

**Post-Lab Questionnaire**

**1. What is your full name?**

_____________________________________________________________

**2. This scale consists of a number of words that describe different feelings and emotions. Read each item and indicate to what extent you feel this way right now, that is, at the present moment (select one per row):**

|  | **Very slightly or not at all** | **A little** | **Moderately** | **Quite a bit** | **Extremely** |
| --- | --- | --- | --- | --- | --- |
| **1. Interested** | ☐ | ☐ | ☐ | ☐ | ☐ |
| **2. Distressed** | ☐ | ☐ | ☐ | ☐ | ☐ |
| **3. Excited** | ☐ | ☐ | ☐ | ☐ | ☐ |
| **4. Upset** | ☐ | ☐ | ☐ | ☐ | ☐ |
| **5. Strong** | ☐ | ☐ | ☐ | ☐ | ☐ |
| **6. Guilty** | ☐ | ☐ | ☐ | ☐ | ☐ |
| **7. Scared** | ☐ | ☐ | ☐ | ☐ | ☐ |
| **8. Hostile** | ☐ | ☐ | ☐ | ☐ | ☐ |
| **9. Enthusiastic** | ☐ | ☐ | ☐ | ☐ | ☐ |
| **10. Proud** | ☐ | ☐ | ☐ | ☐ | ☐ |
| **11. Irritable** | ☐ | ☐ | ☐ | ☐ | ☐ |
| **12. Alert** | ☐ | ☐ | ☐ | ☐ | ☐ |
| **13. Ashamed** | ☐ | ☐ | ☐ | ☐ | ☐ |
| **14. Inspired** | ☐ | ☐ | ☐ | ☐ | ☐ |
| **15. Nervous** | ☐ | ☐ | ☐ | ☐ | ☐ |
| **16. Determined** | ☐ | ☐ | ☐ | ☐ | ☐ |
| **17. Attentive** | ☐ | ☐ | ☐ | ☐ | ☐ |
| **18. Jittery** | ☐ | ☐ | ☐ | ☐ | ☐ |
| **19. Active** | ☐ | ☐ | ☐ | ☐ | ☐ |
| **20. Afraid** | ☐ | ☐ | ☐ | ☐ | ☐ |

**3. For the purposes of this question, refer to this dog fear scale:**

| **Fear score** | **Posture** | **Avoidance** | **Other Behaviors** |
| --- | --- | --- | --- |
| **Score 0** | Head normal or high, ears forward, tail high or breed-specific position | No escape attempts | None |
| **Score 1** | Head normal or high, ears forward or slightly back, tail body-height or slightly lowered | No escape attempts | 1–2 subtle behaviors: Yawning, lip licking, body shaking, vocalizing, trembling |
| **Score 2** | Head normal or low, ears sideways, tail lowered (still or wagging) | Minor attempts to escape/retreat/hide; few steps backward/away | Several behaviors: Yawning, lip licking, trembling, body shaking, vocalizing |
| **Score 3** | Head somewhat low, ears down/slightly back, tail tucked between legs | Moderate escape/retreat/hide attempts; several steps backward/away | Several behaviors: Yawning, lip licking, trembling, body shaking, vocalizing |
| **Score 4** | Head very low, ears pinned back, tail fully tucked between bent hind legs | Vigorous/constant escape, retreat or hide attempts | Several behaviors: Yawning, lip licking, trembling, body shaking, vocalizing |

**How would you rate the dog patient’s overall level of fear during the physical examination? (select one)**

- Score 0
- Score 1
- Score 2
- Score 3
- Score 4
