# Supplementary material for: Veterinary student wellbeing and its relationship with dog handling and fear responses during a clinical examination laboratory
Source: Front Vet Sci. 2026 Aug 20;13:1910028. doi: 10.3389/fvets.2026.1910028 (PMC13538008; doi:10.3389/fvets.2026.1910028)
Supplement: Supplementary file 2 [file Supplementary_file_2.DOCX]

Supplementary Material

**Table 1.** Veterinary students’ (n = 66) reported level of comfort handling calm, fearful, and aggressive dogs during routine examinations.

| Dog demeanor | Very uncomfortable | Slightly uncomfortable | Neither comfortable nor uncomfortable | Slightly comfortable | Very comfortable |
| --- | --- | --- | --- | --- | --- |
| Calm dog | 4 (6.1) | 0 (0) | 0 (0) | 0 (0) | 62 (93.9) |
| Fearful dog | 3 (4.5) | 1 (1.5) | 5 (7.6) | 30 (45.5) | 27 (40.1) |
| Aggressive dog | 3 (4.5) | 14 (21.2) | 17 (25.8) | 22 (33.3) | 10 (15.2) |

**Table 2.** Veterinary students’ (n = 65) reported level of comfort handling different dog sizes and animal species.

|  | Strongly agree | Agree | Neutral | Disagree | Strongly disagree |
| --- | --- | --- | --- | --- | --- |
| I am more comfortable handling medium/large dogs (e.g., ≥35lbs compared to small dogs (e.g., <35lbs) | 33 (50.8) | 15 (23.1) | 14 (21.5) | 2 (3.1) | 1 (1.5) |
| I am more comfortable handling cats compared to dogs | 4 (6.2) | 13 (20.0) | 23 (35.4) | 22 (33.9) | 3 (4.6) |
| I am more comfortable handling livestock (e.g., cattle, horses) compared to dogs | 19 (29.2) | 14 (21.5) | 14 (21.5) | 14 (21.5) | 4 (6.2) |

**Table 3.** Veterinary student mental well-being and affective state reported before and after the clinical lab; all examiner and handler students (n = 66) and handlers (n = 33).

| Group | Measure | Pre-lab  mean ± SE (range) | Post-lab  mean ± SD (range) |
| --- | --- | --- | --- |
| Examiners and handlers | WHO-5 | 68.5 ± 19.3 (16–100) | − |
|  | PA | 34.2 ± 8.6 (15–50) | 34.5 ± 9.5 (12–50) |
|  | NA | 16.5 ± 5.5 (10–34) | 14.2 ± 6.0 (10–40) |
| Handlers only | WHO-5 | 70.5 ± 16.4 (36–100) | − |
|  | PA | 34.7 ± 7.8 (21–50) | 34.8 ± 8.5 (14–46) |
|  | NA | 15.2 ± 4.5 (11–33) | 12.3 ± 3.3 (10–25) |
